# Congenital Epidermoid Cyst Results in Muscle Fusion Defect in the Upper Lip

**DOI:** 10.1155/2014/540910

**Published:** 2014-12-31

**Authors:** Fatih Dogan, Ibrahim Hakan Bucak

**Affiliations:** ^1^Department of Plastic Surgery, Adıyaman University School of Medicine, 02200 Adıyaman, Turkey; ^2^Department of Pediatrics, Adıyaman University School of Medicine, 02200 Adıyaman, Turkey

## Abstract

Epidermoid cysts are rarely detected malformations in the oral cavity. Their development sites are the sublingual, submaxillary, and submandibular spaces. In this paper, we report a three-month-old infant who was admitted to our hospital due upper lip swelling. Magnetic resonance imaging showed that she had a two-centimeter cystic lesion and fusion defects of orbicularis oris muscle. The cyst was surgically removed and histopathological diagnosis was “epidermoid cyst.” In recent literature, we could not find reports related to orbicularis oris muscle fusion defects because of epidermoid cyst.

## 1. Introduction

Dermoid or epidermal cysts arise from aberrant localization of epithelial component of ectodermal tissue in fetal life or aberrant inoculation of epithelial tissue after trauma or surgery [1]. These cysts can be encountered in any part of the body, but their prevalence is approximately 1.6–6.9% in the head-neck region and about 0.01% in the oral cavity [1–3]. In the literature, there are case reports comprising patients that presented with pain, speech disorder, or respiratory distress due to epidermoid cyst in the oral cavity, lower lip, or upper lip [4–7]. Herein, we presented a case that could be defined as microform cleft lift that presented itself only with loss of muscle fusion due to congenital epidermal cyst in the upper lip, which has not been encountered before.

## 2. Case Report

A 3-month-old infant was brought to our clinic with swelling in the upper lip, which was present at birth. The patient's parents had no remarkable disease in their medical history and the patient's mother suffered from no disease and she did not have any medication during her pregnancy. The infant was healthy with no history of trauma or surgery.

**Figure 1:**
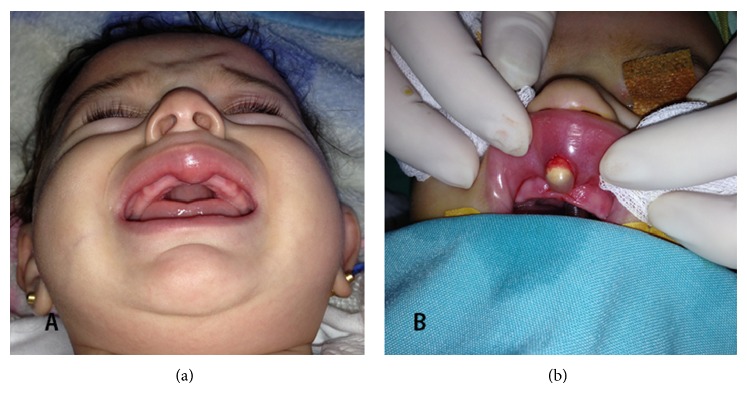
(a) Infant patient with swelling in the upper lip. Lesion located under the philtral columns in the gingiva-buccal mucosal area of the upper lip. (b) Perioperative surgical excision of the lesion.

**Figure 2:**
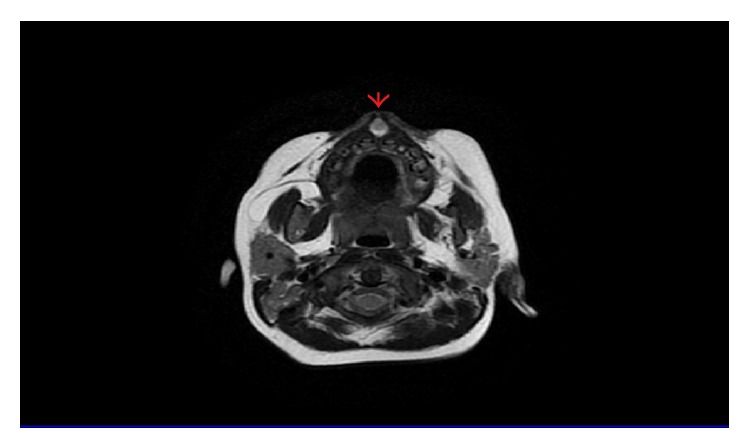
Magnetic resonance imaging shows the lesion that prevented the fusion of orbicularis oris muscles in the midline of the upper lip.

When the infant cried and smiled, smiling deformity appeared. Intraoral examination revealed nearly 2 cm swelling located under the philtral columns in the gingiva-buccal mucosal area of the upper lip (Figure 1(a)). There was no sign of inflammation, and oral mucosa was in normal color and texture. Sinus tract was seen between philtral columns over the skin of upper lip. Magnetic resonance imaging, which was performed for differential diagnosis, demonstrated nearly 2 cm cystic lesion attached to the skin and prevented the fusion of orbicularis oris muscles in the midline of the upper lip (Figure 2).

When the patient was 4 months old, the lesion was dissected from surrounding tissues through a midline incision made on the oral mucosa of the upper lip after performing orotracheal intubation under general anesthesia, and sinus tract was completely excised via subepidermal excision (Figure 1(b)). Fusion defect is also observed in the orbicularis oris muscle of the upper lip. Subsequently, orbicularis oris muscle was repaired in the midline following a minimal dissection. No complication developed in the postoperative period.

**Figure 3:**
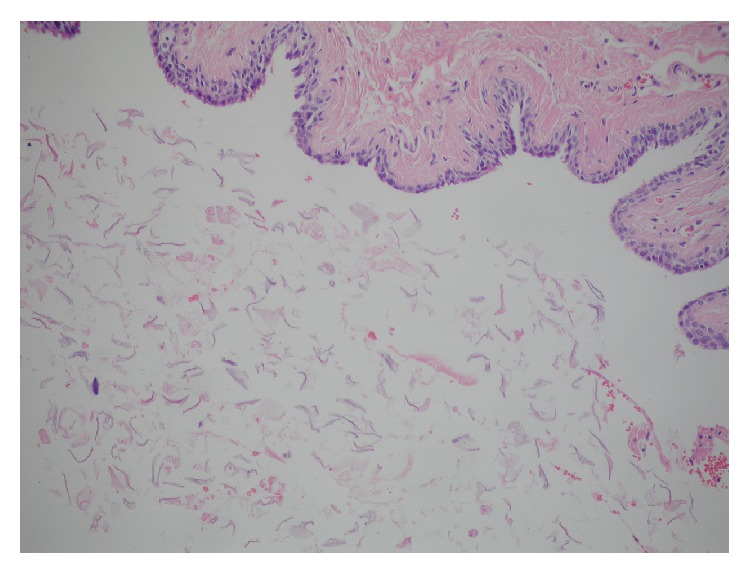
Histopathological section showing a cyst lined by keratinized squamous epithelium, cavity filled with lamellated keratin.

Excision biopsy and histopathological examination of the resected specimen showed a cyst lined by stratified squamous epithelium filled with lamellated keratin (Figure 3). No skin appendages were seen. Thus, histopathological diagnosis of epidermoid cyst was confirmed.

## 3. Discussion

The etiology of epidermal cysts and dermoid cysts is unclear. Clinical features of craniofacial dermoid and epidermoid cysts are presented in varied ways, including infection, asymptomatic puncti, or seizure secondary to intracranial invasion. Many theories have been suggested [8, 9]. It has been reported that these cysts might occur due to aberrant localization of epithelial component of ectodermal tissue in the fetal period or aberrant inoculation of epithelial tissue after trauma or surgery. Epidermal cyst lesions, which are considered to be congenital, appear in the 4–10 weeks of fetal period in head-neck region, most frequently in the fusion areas of branchial arches, but may also be located in ectopic areas. During this time the nasal placodes invaginate to form the medial nasal processes, which in turn form the intermaxillary processes. Fusion of the intermaxillary processes by the 10th week of gestation and entrapment of neuroectoderm at the midline of these processes may have resulted in the frenulum epidermoid as reported in this case. In our case, congenital swelling in that region with muscle fusion defect observed on magnetic resonance imaging suggests that the defect absolutely developed in fetal life at the time of fusion.

To the best of our knowledge, there is no similar case in the literature. Although there are pediatric cases in the literature with epidermal cyst lesions in the sublingual region, gingiva, palate, and uvula, many of them had history of trauma or surgical intervention [1, 8, 10]. Total excision is the basic treatment for intraoral epidermal cystic lesions since needle aspiration or fenestration might enhance infection, pain, and complaints after treatment.

In recent literature, we could find no reports related to orbicularis oris muscle fusion defects because of epidermoid cyst. Due to muscle fusion defect in our case, the best treatment timing for the patient was planned mainly based on the repair time of cleft lip deformity. Thus, lip muscle could be repaired at appropriate time in the same session with the excision of lesion.
